# Pensions for Hospital Workers.—I

**Published:** 1890-11-22

**Authors:** 


### Passing Topics.

### PENSIONS FOR HOSPITAL WORKERS.—I.

To everybody dependent upon the work of hand or brain for the provision of all things necessary to existence, this serious and perhaps unwelcome question must from time to time intrude itself upon attention: What am I to do when failure of health or the feebleness of age come upon me and I can work no more? Especially must this question come home to every thoughtful member of that large class whose employment precludes all hope of a substantial margin of income over expenditure.

In such circumstances something more scientific than mere saving is needed to banish that acute solicitude concerning the future, which is to some a perpetual nightmare, disturbing lives which might have been passed in contentment and embittering labours otherwise not unsweet.

The National Pension Fund for Nurses and Officials is an organization devoted to the interests of a body of workers whose case aptly illustrates these reflections, and if the founder had put his hand to no other labour in the hospital field, he would yet have deserved well of the hospital community for a potent effort in the cause of true philanthropy. A perusal of the report issued shows that the progress of the National Pension Fund has been rapid, a fact due, as we may believe, to its own inherent recommendations. Considerable interest has been aroused in its operations; it has received a mark of high favour at the hands of the Prince and Princess of Wales, and the results already achieved are such as fully justify the legitimate pride of the promoters, and lead to the belief that this Fund is destined to a very important future.

The National Pension Fund is not a charity, but its objects are none the less beneficent. It is directly concerned in the encouragement of thrift among a class of workers who cannot be negligent with safety, and it possesses this marked advantage and recommendation over a society established upon a purely business basis; that its managers have no thoughts of commercial success, but are actuated by a single-minded desire to benefit those in whose behalf the fund has been founded.

The fund is stated to be conducted upon "mutual" principles, but it is mutual, in a sense, differing considerably, and all to the advantage of the insurer, from that employed in respect of a company where capital comes in for a share of the profits. In this case the funds are administered wholly for the benefit of the policy-holders, and the profits and increments, from whatever source derived, will be applied to increasing the pensions.

A Bonus Fund, the interest upon which will go to swell the pensions, has been started, and with a munificence calling for especial acknowledgment, donors have come forward with offerings amounting in all to £40,000. This sum is a free gift to our hospital nurses, for to them the advantages of the Bonus Fund are limited, and there is none but will rejoice that they should receive so splendid a token of sympathy and appreciation.

These are the general outlines of the scheme: In a subsequent paper we will deal more precisely with its details.

Agricola.

